# Review on Usage of Vancomycin in Livestock and Humans: Maintaining Its Efficacy, Prevention of Resistance and Alternative Therapy

**DOI:** 10.3390/vetsci4010006

**Published:** 2017-01-26

**Authors:** Panditharathnalage Nishantha Kumara Wijesekara, Wikum Widuranga Kumbukgolla, Jayaweera Arachchige Asela Sampath Jayaweera, Diwan Rawat

**Affiliations:** 1 Human Recourse Department, University Grants Commission, 20, Ward Place, Colombo 07 10000, Sri Lanka; nkwijesekara@gmail.com; 2 Department of Biochemistry, Faculty of Medicine and Allied Sciences, Rajarata University Mihintale, Mihintale 50008, Sri Lanka; kumbukgolla@yahoo.com; 3 Department of Microbiology, Faculty of Medicine and Allied Sciences, Rajarata University Mihintale, Mihintale 50008, Sri Lanka; 4 Department of Chemistry, University of Delhi, Delhi 110007, India; dsrawat@chemistry.du.ac.in

**Keywords:** vancomycin, broad view, veterinary use at a glance, rational use, alternatives

## Abstract

Vancomycin is one of the “last-line” classes of antibiotics used in the treatment of life-threatening infections caused by Gram-positive bacteria. Even though vancomycin was discovered in the 1950s, it was widely used after the 1980s for the treatment of infections caused by methicillin-resistant *Staphylococci*, as the prevalence of these strains were increased. However, it is currently evident that vancomycin-resistant *Staphylococcus aureus* and vancomycin-resistant *Enterococci* have developed for various reasons, including the use of avaparcin—an analog of vancomycin—as a feed additive in livestock. Therefore, prophylactic and empiric use of antibiotics and their analogues need to be minimized. Herein we discuss the rational use of vancomycin in treating humans, horses, farm animals, and pet animals such as dogs, cats, and rabbits. In present day context, more attention should be paid to the prevention of the emergence of resistance to antibiotics in order to maintain their efficacy. In order to prevent emergence of resistance, proper guidance for the responsible use of antimicrobials is indispensable. Therefore, almost all stakeholders who use antibiotics should have an in-depth understanding of the antibiotic that they use. As such, it is imperative to be aware of the important aspects of vancomycin. In the present review, efforts have been made to discuss the pharmacokinetics and pharmacodynamics, indications, emergence of resistance, control of resistance, adverse effects, and alternative therapy for vancomycin.

## 1. Introduction

Vancomycin was first discovered from a soil sample in the interior jungle of Borneo in the 1950s, and its usage was very limited due to the presence of impurities that caused toxicities in the earlier preparations. However, the use of vancomycin was reconsidered after the emergence of methicillin-resistant *Staphylococci* in the 1970s, and its usage increased from the 1980s after purer preparations were made in late 1970s [1]. Now, vancomycin has become the most common injectable drug of choice to treat methicillin-resistant *Staphylococci* species and drug resistant *Enterococcus* species [2].

Vancomycin exhibits bactericidal activity by inhibiting the cell wall synthesis against aerobic and anaerobic Gram-positive bacteria [3]. Vancomycin is active against most strains of Clostridia, almost all strains of *Staphylococcus aureus* (including those that produce β-lactamases and methicillin resistant strains), coagulase-negative Staphylococci, and Viridans group Staphylococci and Enterococci. Vancomycin is not effective against Gram-negative bacteria [4]. Vancomycin is one of the antibiotics of last resort, used only after treatment with other antibiotics has failed in the treatment of life-threatening infections by Gram-positive bacteria. Even though vancomycin has great potential in treating infections in animals, the use of vancomycin in veterinary medicine is limited because it is expensive and requires continuous intravenous infusion [5].

Available dosage forms of vancomycin are 500 mg, 1 g, 5 g, and 10 g vials for injections. Powdered vancomycin is reconstituted in sterile water, which results in a dark-colored solution, and it is further diluted in 5% dextrose or saline when it is administered. The reconstituted solution is stable for 14 days either at room temperature or in a refrigerator. Additionally, 125 mg and 250 mg vancomycin tablets are available for oral administration [6].

Improper use of antibiotics is largely responsible for the microbial drug resistance problems. Therefore, the people who use antibiotics in animals and humans must be vigilant about the adverse effects and the proper doses—especially in case of last-line antibiotics such as vancomycin. In this review, efforts have been made to illustrate the usage of vancomycin in animals and humans; however, the review shows areas that need more clinical trials on animal models, as little information is available for its use in animals. The limited use of vancomycin in animals is due to various reasons, such as it is a last line of antibiotic in humans, it is inconvenient to administer in animals, the emergence of vancomycin-resistant *Enterococci*, and the threat of spread of vancomycin-resistant genes to other gram-positive organisms. However, vancomycin is a valuable drug of choice for the treatment of infections of animals that are caused by multi drug resistant *Enterococci* and *Staphylococci* species [7].

## 2. Pharmacokinetics and Pharmacodynamics of Vancomycin

**Figure 1:**
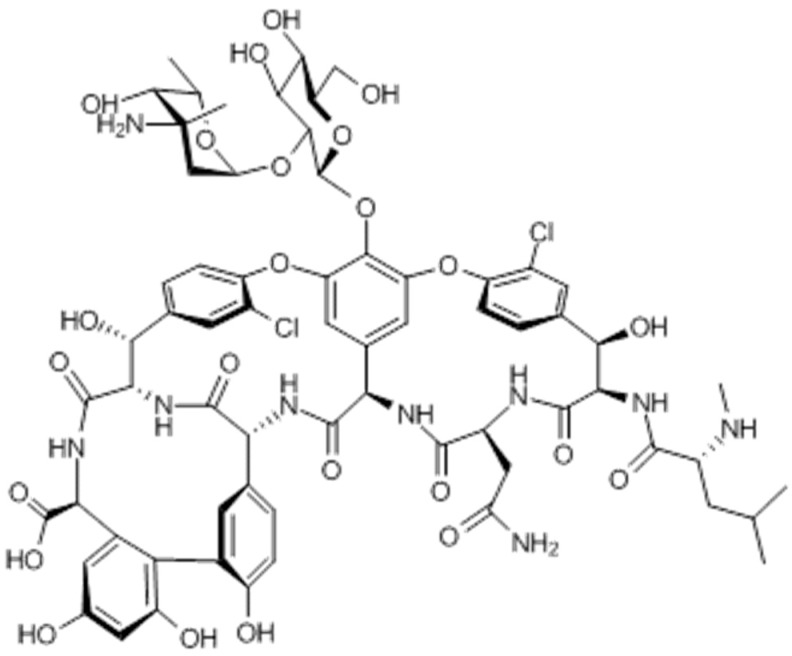
Chemical structure of vancomycin [12].

Vancomycin is a large glycopeptide compound with a molecular weight of 1448 Da which inhibits a late stage in bacterial cell wall peptidoglycan synthesis [8,9]. Amino acids present in vancomycin are synthesized, joined together, and cross-linked to assemble vancomycin [10]. The chemical structure of vancomycin is displayed in Figure 1. The three-dimensional structure of vancomycin contains a cleft that fits by hydrogen bonding with the peptides of a highly specific configuration of l-alanyl-d-alanyl-d-alanyl which is found only in bacterial cell walls; therefore, vancomycin is selectively toxic by forming stable complexes [11].

The factors that affect the activity of vancomycin are its tissue distribution, its protein-binding, inoculum size, and resistant organisms. The volume of distribution in humans is 0.4–1 L/kg; in dogs, 0.4–5.5 L/kg [13]. The binding of vancomycin to protein has a range from 10% to 50%. A 1–8-fold increase in the minimum inhibitory concentration (MIC) has been shown in several in vitro assessments as a result of the presence of albumin, whereas the presence of serum has had a more variable effect [14,15]. It is evident in an in vitro pharmacodynamic model that the time taken to kill is longer when the inoculum size is high (9.5 log_10_ CFU/g) compared to a moderate inoculum (5.5 log_10_ CFU/g): 48 versus 72 h, respectively, for both the methicillin sensitive *Staphylococci* and methicillin-resistant *Staphylococci* organisms isolated from human patients [16,17].

Vancomycin penetrates into most body spaces, and the penetrability is dependent on the degree of inflammation present. The concentration of vancomycin in different body spaces is different [18]. The inflamed meninges improve the penetration of vancomycin into the cerebral spinal fluid, with reported concentrations of 6.4–11.1 mg/L, whereas uninflamed meninges have resulted in low concentrations of 0–3.45 mg/L in humans [19]. Furthermore, it has been shown in a rabbit model that a high concentration of vancomycin is present in the cerebral spinal fluid of inflamed meninges [20]. Therapeutic concentrations of vancomycin in ascitic, pericardial, pleural, and synovial fluids are greater than 2.5 mg/L in humans [21].

More than 80% and 50% of a vancomycin dose is excreted unchanged in the urine (mostly by way of glomerular filtration) within 24 h after administration in humans and dogs, respectively, and the concentration of vancomycin in liver tissue and bile is below detectable levels. Vancomycin has a distribution phase of 30 min to 1 h. The half-life of vancomycin in patients with normal creatinine clearance in humans is about 6 h; dogs, 2 h; horses, 3 h [22,23].

## 3. Therapeutic Indications of Vancomycin

As vancomycin is a last resort antibiotic in humans, its use in humans and animals is limited. That may be the reason for the scarcity of available reference materials on the use of vancomycin in livestock. However, vancomycin would be a compulsory drug of choice in valuable animals such as breeding animals in similar indications as humans. Even though the reference material for following indications mainly deal with human medical conditions, vancomycin can be used in those conditions in animals, as vancomycin has been tested in lab animals and it is suggested for clinical trials in animals so as to establish proper guidelines for veterinary clinical practice.

A significant reduction of the number of colony forming units of *Staphylococcus aureus* in mouse blood was observed following vancomycin therapy [11]. It was shown that 165 *Enterococcus* strains isolated from dogs were sensitive to vancomycin, despite the fact that they show a high frequency of resistance to erythromycin, tetracycline, rifampicin, and enrofloxacin [24].

Vancomycin is given to humans by the intravenous route in prophylaxis when there is a high risk of methicillin-resistant staphylococci, and for treatment of endocarditis, osteomyelitis, acute bacterial prostatitis, and other serious infections caused by Gram-positive cocci [25]. Human patients with normal renal function should receive a loading dosage of 25–30 mg of vancomycin per kg intravenously over 1 h, followed by a regular dose every 12 h [26,27]. However, the practical dosing intervals can be 8, 12, 24, and 48 h based on the creatinine clearance of the patient [28].

Currently, dosages of vancomycin for administration in animals are highly empirical. In general, intravenous administration of vancomycin (diluted in 200 mL of 5% dextrose) for animals is at a dose rate of 20 mg/kg over a 1-h period at 12 h intervals [29]. More specifically, the vancomycin dosage for horses is 4.3–7.5 mg/kg in 8 h intervals, and for dogs it is 15 mg/kg in 6 h intervals, intravenously over a 1-h period. For dogs, a loading dose of 3.5 mg/kg and constant rate infusion of 1.5 mg/kg/h can be administered [2,30,31]. Vancomycin can be used to treat infections caused by erythromycin- and rifampin-resistant *Rhodococcus equi* in young horses [32,33]. In view of the above, it is apparent that the intravenous dose rate of vancomycin for horses falls within the range of dose recommended for humans. In healthy horses, a therapeutic concentration of vancomycin can be reached and maintained in synovial fluid after IV administration [34]. Vancomycin is a good therapeutic option in children having hematogenous osteomyelitis caused by methicillin-resistant *Staphylococcus aureus* (MRSA). In contrast, a case series pointed out that the potential drawback of vancomycin therapy in foals in severe osseous or physeal lesions (multiple physeal abscesses) or severe joint disease (fibrinous septic arthritis) may have a limited drug delivery to the site(s) of infection [35]. However, it is necessary to execute clinical trials to establish exact dose rates for animals, and it is desirable to measure creatinine clearance in animals to decide upon practical dosing intervals.

Vancomycin is administered locally to treat localized joint or bone infection in horses by regional limb perfusion of 300 mg diluted in a 0.5% solution [2,36]. Vancomycin is given to lobsters suffering from gaffkaemia due to Gram-positive bacteria, by way of giving injection in to abdominal sinus at a dose rate of 25 mg/kg [37].

Vancomycin is used in humans by mouth, at a dosage of 125 mg every 6 h for 7 to 10 days in the treatment of pseudomembranous colitis caused by overgrowth of *Clostridium difficile. Clostridium difficile* also causes *Clostridium difficile*-associated diseases in swine, calf, and horses [38,39]. The empirical dose rate for oral administration in animals is 5–10 mg/kg every 12 h. Vancomycin can be used orally for *Clostridium perfringens* enteritis and *Clostridium spiroforme* enteritis in rabbits, or *Clostridium difficile* in hamsters [40] and other species, including horses. It has been clinically proven that vancomycin can be used to treat for cholangio-hepatitis caused by a beta-lactam-resistant *Enterococcus* in cats at a dose rate of 12–15 mg/kg/h intravenously [41]. Thus, the exact dosage for those indications also should be established with proper dosage intervals. For the treatment of peritonitis in humans, vancomycin is added to dialysis fluid [42].

Vancomycin can be more effective in combination with other antibiotics for cases in which vancomycin alone is ineffective. The synergistic action of vancomycin either with gentamicin or streptomycin helps to kill susceptible strains of enterococci [43]. It has been demonstrated in humans that vancomycin is better than trimethoprim-sulfamethoxazole in efficacy and safety in treating staphylococcal infections [44]. As such, it is necessary to conduct animal clinical trials investigating the synergistic action of vancomycin with other drugs. This phenomenon has been demonstrated in vitro using a modified disk diffusion test to elucidate the synergistic action of vancomycin with ceftriaxone, ceftazidime, cefpodoxime, and amoxicillin-clavulanate against methicillin-resistant staphylococci. It was shown using a rabbit model that the combination of vancomycin with nafcillin has greater efficacy against vancomycin intermediate-susceptible *S. aureus* in aortic valvular vegetation and renal abscesses than by either treatment alone [45].

## 4. Emergence of Resistance to Vancomycin

Antibiotic use either as therapy, in the prevention of bacterial diseases, or as performance enhancers has resulted in antibiotic-resistant micro-organisms in pathogens and among bacteria of the endogenous microflora of animals. Antibiotic-resistant bacteria present in animals can be transmitted to humans via contact or via the food chain. Furthermore, resistance genes of animal bacteria can be transferred to human pathogens in the intestinal flora of humans [37].

The development of intermediate and high levels of resistance to vancomycin for *Staphylococcus aureus* was reported for the first time in Japan in 1997 [46]. According to guidelines of the Clinical Laboratory Standards Institute, susceptibility break points of vancomycin are ≤4 mcg/mL for *Enterococcus*, ≤1 mcg/mL for *Streptococcus*, and ≤4 mcg/mL for *Staphylococcus.* However, in 2006, the vancomycin MIC breakpoints for *S. aureus* were lowered to 2 µg/mL for “susceptible”, 4–8 µg/mL for “intermediate”, and 16 µg/mL for “resistant” [47]. Enterococci should be regularly tested in vitro for susceptibility to vancomycin for determination of MIC. Enterococci are deemed susceptible to vancomycin if MICs are ≤4 μg/mL; they are considered as intermediate level resistance to vancomycin if MICs are 8 to 16 μg/mL; and as complete resistance to vancomycin if MICs are >16 μg/mL [23].

Mortality of people increases when the MRSA bacteremia was caused by strains with a high vancomycin resistance (MIC > 1 µg/mL) and when it was treated empirically either with inappropriate antibiotic or vancomycin [48,49,50]. Avoparcin (a vancomycin analog) is a glycopeptide antibiotic that can suppress the growth of Gram-positive bacteria, and it has been used in livestock feed for growth promotion in broiler chickens, growing pigs, calves, and beef cattle. Avoparcin has also been used for preventing necrotic enteritis in poultry. In countries where avoparcin was used for the above purposes, it was evident that vancomycin-resistant enterococci (VRE) are commonly found in the commensal microbiota of food animals and in the meat from these animals. The presence of VRE in the commensal microbiota of healthy humans has been observed despite the very low usage of vancomycin in hospitals [37].

The harmless commensal of enterococci have evolved over the years to opportunistic pathogens mainly causing nosocomial infections (hospital acquired infections). The development of VRE is one of the consequences of this phenomenon. The most clinically important bacterial species with a resistance gene is *Enterococcus faecium* with vanA type vancomycin resistance, which is the most common VRE variant among farm animals, where avoparcin is widely used for growth promotion. When the use of avoparcin was discontinued, the prevalence of VRE among farm animals reduced [51,52,53,54].

## 5. Control of Resistance for Vancomycin

The development of preventive strategies to limit existing resistance and to avoid the emergence of resistant bacteria is of paramount importance in maintaining the efficacy of antibiotics in both human medicine and veterinary medicine. Therefore, understanding the epidemiology of antibacterial resistance will enable us to develop preventive strategies to limit existing resistance and to avoid the emergence of new strains of resistant bacteria [55,56].

In order to control emergence of resistance, hygienic measures to prevent cross contamination and a decrease in the usage of antibiotics are desirable. The reduction of the need for antibiotics is the best possible way of controlling resistance in large groups of animals. This can be accomplished by proper vaccination against infectious diseases, the adoption of good hygienic practice in animal husbandry, stopping the use of antibiotics as feed additives for growth promotion in animals bred as a food source, the appropriate use of antibiotics for food animals, and the development of guidelines, codes of practice, and policies on the appropriate use of antibiotics. Farm workers and owners of pets being treated with antibiotics need to pay attention to hygiene during and after handling treated animals [37].

In an infection caused by MRSA strains with elevated vancomycin MIC (2 µg/mL) needs elevated vancomycin dosing to achieve a serum concentration of vancomycin greater than 15 µg/mL. To get such concentrations, it is required to increase the recommended dosage, which may cause toxicities. Hence, a combination or alternative therapy should be considered for such infections [57,58]. The calculation of doses of vancomycin based on MIC values relevant to the infected bacterial strain and physiological condition of the animal is important [59]. Vancomycin usage in animals should be restricted to infections which respond only to vancomycin and for which there are no other reasonable alternatives; when it is used in animals, it should be given at proper dosage, proper dosage interval, and proper duration of treatment [32].

## 6. Adverse Effect of Vancomycin

Although there is a little information on toxicity in animals, there is a high possibility in animals for the following adverse effects, which are evident in human clinical trials [5]. It is reported that vancomycin administration may lead to fatal enterotoxaemia in guinea pigs [42].

Prolonged intravenous use of vancomycin may cause neutropenia, thrombophlebitis, rash, fever, anemia, thrombocytopenia, and ototoxic reactions in humans and animals. Vancomycin should be administered intravenously in diluted form, because it is highly irritable for the tissues. It may cause local phlebitis at the site of injection in animals [6,8]. Vancomycin should be infused for ≥1 h to reduce the risk of the histamine release-associated “red man” syndrome in humans. It is advised not to administer intravenous rapidly, so as to avoid acute adverse reaction in animals [2]. The major drawback of vancomycin usage is auditory damage in humans; however, tinnitus and deafness might improve once the treatment is ceased. In addition to that, nausea, chills, phlebitis, severe hypotension, wheezing, dyspnoea, urticaria, and pruritus have been observed with the treatment of vancomycin in humans [60,61,62,63]. In some instances, neutropenia was detected with prolonged therapy [64].

There is a potential for nephrotoxicity and ototoxicity with vancomycin in animals [65]. Toxicities are minimal in vancomycin monotherapy at conventional dosages of 1 g (15 mg/kg) every 12 h in humans [66]. However, increased incidence of nephrotoxicity has been established with doses of 4 g/day or higher. As a result of elevated dosage, serum concentrations may increase, which may lead to toxicity [67,68,69,70]. Vancomycin increases the risk of nephrotoxicity in humans with drugs such as amphotericin, capreomycin, cyclosporine, cisplatin, colistimethate, polymyxins, and tacrolimus [42]. There are veterinary indications for the above drugs, such as amphotericin used in systemic fungal infections; cyclosporine used in atopy; cisplatin used in osteosarcoma; polymyxins used in gut infections; and tacrolimus used in keratoconjunctivitis sicca in small animals. Depending on the circumstances such as concurrent malignancy or fungal infection in association with MRSA infection, veterinarians would combine such drugs with vancomycin [71,72,30,31]. Therefore, it is desirable to investigate the adverse effects when the above drugs are administered concurrently with vancomycin in animals.

## 7. Alternative Therapy for Vancomycin

Alternative therapies should be considered for humans with *S. aureus* infections that show a vancomycin MIC of 2 mg/L or greater [49]. Lysostaphin (an endopeptidase) is more effective than vancomycin in treating methicillin-resistant *Staphylococcus aureus* in a neonatal pup model [15,24].

Oral bacitracin can be considered as an alternative to vancomycin in the treatment of antibiotic-associated pseudomembranous colitis caused by *Clostridium difficile* cytotoxin in animals. Bacitracin is used for bacitracin-sensitive infections in pigs, chicken, and turkeys [65].

Linezolid—an oxazolidinone active against Gram-positive bacteria—is one of the options for vancomycin in the treatment of infections that are caused by antibacterials including methicillin-resistant *Staphylococcus aureus* and vancomycin-resistant enterococci infections in humans. It has been shown in a murine model that linezolid can be used to control *Mycobacterium tuberculosis* infection. Linezolid such as Zyvox is also used in dogs [73]. Moreover, linezolid is used in animals for the treatment of nocardiosis. Therefore, it is necessary to establish efficacy and dose rates for linezolid in animals [74,75]. Prolonged usage and dose less than that recommended may lead to the development of resistance to linezolid. As linezolid is not active against Gram-negative organisms, it must be coupled with another antibacterial agent if the infection involves both Gram-positive and Gram-negative organisms, and this combination should be used for infections only when other treatments are not available [72]. It has been demonstrated in a rat model that linezolid with rifampin or vancomycin with rifampin is effective in an animal model of MRSA foreign body osteomyelitis [76]. Teicoplanin has a similar activity on MRSA with minimal renal toxicity. Further, red man syndrome is less frequent [77]. The therapeutic drug monitoring of vancomycin suggests that an area under the curve to Minimal inhibitory concentration (AUC/MIC) ratio of ≥400 µg·h/mL AUC/MIC ratio of ≥400 is the pharmacodynamic and pharmacokinetic (PK/PD) parameter associated with clinical and bacteriological responses to vancomycin therapy. In contrast in teicoplanin, an AUC_24_/MIC ratio of ≥900 µg·h/mL is required [35].

Other therapeutic options for vancomycin are trimethoprim-sulfamethoxazole, doxycycline, or minocycline either with or without rifampin. Although vancomycin is superior to trimethoprim-sulfamethoxazole in efficacy and safety, trimethoprim-sulfamethoxazole can be given in selected cases of MRSA infection where there is a treatment failure with vancomycin treatment [45]. All of the above drugs are used in animals [65]. Rifampin can be used to treat pneumonic condition in foals caused by *Rhodcoccusequi* at a dose rate of 5 to 10 mg/kg orally at 12 h intervals. Rifampin has been suggested for use in the treatment of atypical bacterial infection in cats [5,65]. Further, the novel anti-MRSA cephalosporin ceftobiprole is in the pipeline. Its efficacy in veterinary medicine needs to be elucidated.

It has been shown in chicks that dietary cell-wall preparation of *Enterococcus faecalis* strain EC-12 can be used to stimulate the gut immune system and to reinforce the immune reaction against the vancomycin-resistant enterococci [78].

## 8. Conclusions

As vancomycin is a last resort antibiotic where other antibiotics cannot be used, it is essential to maintain efficacy of vancomycin for treating humans, pet animals and livestock species. In order to achieve the above-mentioned objective, vancomycin should be used only in instances where it is necessary to use the proper dosing, dosing interval, and appropriate duration of treatment based on MIC values of the disease-causing agent, physiological condition of the animal, and combination of antibiotics (where appropriate). There is scant research on the use of vancomycin in animals. According to the facts discussed in this review, it is necessary to establish novel parameters for the clinical usage of vancomycin in treating animals, by conducting animal clinical trials in order to minimize the emerging antibiotic resistance of micro-organisms against vancomycin in animals and transferring those organisms to humans. Some countries have prohibited the use of vancomycin analogues in animal food additives, which seems to be a late decision because vancomycin-resistant genes were already evolved before the bans were instituted. Therefore, vigilance in monitoring antibiotic resistance is useful to prevent such incidents in the future.
